# Efficiently Classifying Lung Sounds through Depthwise Separable CNN Models with Fused STFT and MFCC Features

**DOI:** 10.3390/diagnostics11040732

**Published:** 2021-04-20

**Authors:** Shing-Yun Jung, Chia-Hung Liao, Yu-Sheng Wu, Shyan-Ming Yuan, Chuen-Tsai Sun

**Affiliations:** 1 Department of Computer Science, National Chiao Tung University, Hsinchu 300, Taiwan; aiallen.cs07g@nctu.edu.tw (C.-H.L.); mauoreo.cs07g@nctu.edu.tw (Y.-S.W.); ctsun@cs.nctu.edu.tw (C.-T.S.); 2 Department of Computer Science, National Yang Ming Chiao Tung University, Hsinchu 300, Taiwan

**Keywords:** lung sounds, convolutional neural network, feature extraction, automatic auscultations, depthwise separable convolution

## Abstract

Lung sounds remain vital in clinical diagnosis as they reveal associations with pulmonary pathologies. With COVID-19 spreading across the world, it has become more pressing for medical professionals to better leverage artificial intelligence for faster and more accurate lung auscultation. This research aims to propose a feature engineering process that extracts the dedicated features for the depthwise separable convolution neural network (DS-CNN) to classify lung sounds accurately and efficiently. We extracted a total of three features for the shrunk DS-CNN model: the short-time Fourier-transformed (STFT) feature, the Mel-frequency cepstrum coefficient (MFCC) feature, and the fused features of these two. We observed that while DS-CNN models trained on either the STFT or the MFCC feature achieved an accuracy of 82.27% and 73.02%, respectively, fusing both features led to a higher accuracy of 85.74%. In addition, our method achieved 16 times higher inference speed on an edge device and only 0.45% less accuracy than RespireNet. This finding indicates that the fusion of the STFT and MFCC features and DS-CNN would be a model design for lightweight edge devices to achieve accurate AI-aided detection of lung diseases.

## 1. Introduction

The term lung sounds refers to “all respiratory sounds heard or detected over the chest wall or within the chest” [1]. In clinical practice, pulmonary conditions are diagnosed through lung auscultation, which refers to using a stethoscope for hearing a patient’s lung sounds. Lung auscultation can rapidly and safely rule out severe diseases and diagnose some pulmonary disorders’ flare-ups. Therefore, a stethoscope has been an indispensable medical device for physicians to diagnose lung disorders for centuries. However, recognizing the subtle distinctions among various lung sounds is an acquired skill that requires sufficient training and clinical experience. As COVID-19 sweeps the globe, lung auscultation still stays vital for monitoring confirmed cases [2]. Remote automatic auscultation systems may play a crucial role in lowering infection risks in medical workers. Hence, how artificial intelligence can be leveraged to assist physicians in performing auscultation remotely and accurately has become ever more imperative.

While a variety of lung sound types have been defined by recent research, this paper adopts the classification suggested by Pasterkamp et al. [3]. Lung sounds can be classified into two main categories: normal and adventitious. Normal sounds are audible through the whole inhalation phase till the early exhalation phase. Spectral characteristics show that these normal sounds have peaks with typical frequencies below 100 Hz, and the sound energy steeply decreases between 100 and 200 Hz [4]. Adventitious sounds are the other sounds usually generated by respiratory disorders and are superimposed on normal sounds. Furthermore, adventitious sounds can be classified into two basic categories: continuous and discontinuous. Continuous and discontinuous sounds were termed wheeze and crackle, respectively, in 1957 [5]. In 1977, wheeze and crackle were sub-classified into more classes according to various ranges of pitches [6]. Continuous sounds (wheeze) are typically musical adventitious sounds with frequencies from 80 to 1600 Hz [7]. The term continuous indicates that the sounds last longer than 250 ms [8]. These continuous sounds are caused by the narrowing of the airway caliber [9]. Two factors determine the pitches of these continuous sounds. One is the mass and elasticity of the airway walls, and the other is the velocity of airflow [9]. The pitches of the continuous sounds correspond to the dominant frequencies in the power spectrum. Continuous adventitious sounds can clinically signify obstructive airway diseases such as asthma and chronic obstructive pulmonary disease (COPD) [10]. Discontinuous sounds (crackle) are non-musical, short, explosive sounds that usually last shorter than 20 ms [10]. Those discontinuous sounds are produced because of the abrupt opening or closing of the airways, which are abnormally closed due to the lung’s increased elastic recoil pressure [11]. The frequency of the discontinuous sounds ranges from 100 to 2000 Hz depending on the airways’ diameter [10]. Additionally, the discontinuous sounds can be related to the disease’s process and severity in patients with pneumonia [12,13] and interstitial lung disorders [11]. Huang et al. [14] found that crackles were one of the common abnormal breath sounds detected through COVID-19 patients’ auscultations.

Learning-based algorithms, particularly deep learning algorithms, have been driving the development of remote automatic lung sound auscultation in recent years [15]. A convolution neural network (CNN), one of the deep learning models, can automatically learn abstract features from images [16]. The visual representations of lung sound signals such as spectrograms can be fed into a CNN as image-like features to train lung sound recognition models [17,18,19,20,21,22,23]. Importantly, CNN models require large datasets for training. The International Conference on Biomedical Health Informatics (ICBHI) Scientific Challenge dataset [24], currently the largest public lung sound dataset, collected three types of adventitious and normal lung sounds records from 126 subjects. Several inspiring deep learning-related methods have been proposed on the basis of the ICBHI dataset. Chen et al. [21] proposed an optimized S-transform to generate spectrograms with enhanced frequency-related features and trained a deep residual network with the special spectrograms to classify three types of lung sounds. The deep residual network achieved 98.79% accuracy on the ICBHI dataset. García-Ordás et al. [25] proposed a variational autoencoder (VAE)-based method to address the imbalanced issue of the ICBHI dataset, which even reached a 0.993 F-score. RespireNet [22] has been proposed to break through the data amount limitation of the ICBHI dataset. The authors of RespireNet augmented training datasets by concatenating two sound signals in the same class. This data augmentation technique greatly improved the accuracy of classifying the adventitious sounds. In addition to CNN, the recurrent neural network (RNN) was proposed to predict respiratory anomalies based on the ICBHI dataset [15]. According to those previous studies [21,25], most CNN-based models tend to achieve high accuracy when classifying lung sounds.

However, the standard CNN models require graphics processing units (GPUs) to support the vast convolutional operations. The depthwise separable (DS) convolution is an approach to reduce the computational operations of the standard convolution [26]. These CNN models with DS convolution layers (DS-CNN) then empower those edge devices with no GPUs and limited computational power to achieve higher efficiency for CNN model inference. The development of automatic lung auscultation systems on low-cost hardware devices has drawn a lot of attention [19,27,28]. How to better exploit the value of DS-CNN for developing remote automatic lung auscultation systems still remains to be explored.

This paper aims to propose a feature engineering process to extract the dedicated features for DS-CNN to classify four types of lung sounds: normal, continuous, discontinuous, and unknown. We shrank a DS-CNN model based on MobileNet [29] to save storage space on edge devices. Then we extracted a total of three features for the shrunk DS-CNN model: the short-time Fourier transformed (STFT) feature, the Mel-frequency cepstrum coefficient (MFCC) feature, and the fused features of these two. To evaluate the performance of the extracted features and the shrunk DS-CNN model, we compared the performance in three hierarchical levels of strategy: level 1—feature comparison; level 2—model architecture comparison; and level 3—model performance and inference efficiency comparison. We observed that the model trained on either the STFT feature or the MFCC feature achieved the accuracy of 82.27% and 73.02%, respectively. Importantly, fusing both features led to a higher accuracy of 85.74% in level 1 comparison. In level 2 comparison, the shrunk DS-CNN outperformed other CNN-based architectures in terms of accuracy and number of parameters. In level 3 comparison, our method achieved 16 times higher inference speed on the edge device with a drop of only 0.45% in accuracy compared to RespireNet.

## 2. Materials and Methods

### 2.1. Dataset

The dataset for this research was prepared after preprocessing the acoustic recordings collected by Lin et al. [30]. These WAV format recordings were 15 s long, and the sampling rate was 4 k Hz. The respiratory cycles in the recordings were segmented into clips and independently labeled by experienced respiratory therapists and physicians as one of the four types: normal, continuous, discontinuous, and unknown. The respiratory cycles with inconsistent labels would be further reviewed and discussed by the annotators for consensus labeling. The audio clips were labeled as unknown if the noise in the clinical environment, such as vocals or equipment sounds, was too loud for the experts to label the definite types. The average length of the respiratory cycles in this dataset was 1.25 s. The audio clips shorter than 1.25 s were padded to this average length with zeros. The clips longer than 1.25 s were truncated as well. After labeling and adjustment of the length, our dataset consisted of 3605 normal, 3800 continuous, 3765 discontinuous, and 1521 unknown lung sound audio clips. This dataset was further divided into three sub-datasets: 72% randomly selected samples for model training, 8% for validating, and the remaining 20% for testing.

### 2.2. Feature Engineering

**Figure 1:**
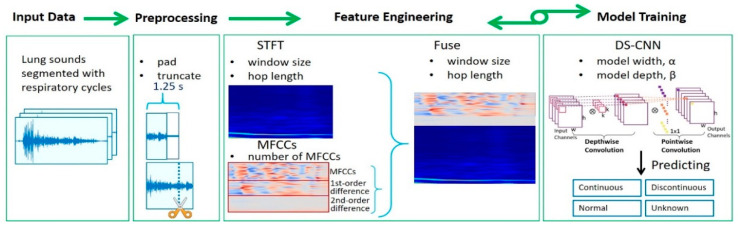
Flowchart of the proposed feature engineering process for depthwise separable convolution neural network (DS-CNN). Before the feature engineering step, each lung sound audio was padded or truncated to 1.25 s-long. A series of parameter combinations were searched in the feature engineering step, including the window size, hop length of the short-time Fourier transformed (STFT) feature, and the number of Mel-frequency cepstrum coefficient (MFCC) features. The DS-CNN model’s width and depth were determined in the model training step. Several DS-CNN models were trained and evaluated to extract the best features. For each feature, we selected the parameter combinations that led the DS-CNN model to achieve the best accuracy.

Our feature engineering process was derived from reference [31]. Fusing of multi-spectrogram features as one new feature has been proposed to improve sound recognition accuracy [31]. A total of three features were extracted. One was the STFT feature, and the second was the MFCC feature. The third feature was extracted by fusing the STFT and MFCC features. The whole feature engineering process is presented in Figure 1.

#### 2.2.1. STFT Feature

STFT transforms only the fast-varying part of the signal, which corresponds to the high-frequency domain, and preserves the low-varying trend in the time domain. For a signal sequence {x(n), n=0,1,… N} of length N, the discrete STFT at the frequency f and the mth short time interval is defined as

(1) $$X\left(f,\;m\right)=\sum_{n=0}^{N-1}x\left(n\right)w\left(n-mR\right)e_{}^{-j2\pi fn},\;\;w\left(n\right)=1\;for\;-\frac{L}{2}\leq n\;\leq \frac{L}{2}\;=0\;\mathrm{otherwise}.$$

**Figure 2:**
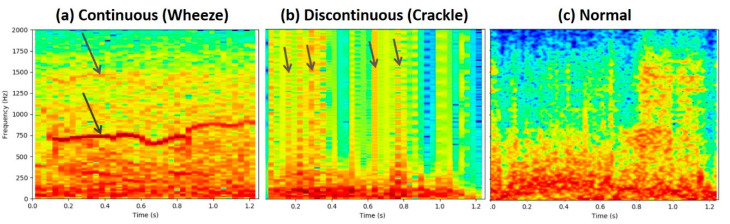
The continuous-sound, discontinuous-sound, and normal-sound spectrograms are shown in (**a**–**c**), respectively. The arrows in (**a**) indicate some peaks of particular frequency domains extending along with the time domain, which implies that the continuous sounds may require high-frequency resolution to extract distinguishable features. The arrows in (**b**) point out that dozens of peaks of particular frequencies go up and down alternatively in a relatively short period along with the time domain, which implies that time resolutions are more relevant to extract recognizable features for the discontinuous sounds. The normal-sound spectrogram (**c**) weighs more in the low-frequency region.

Here *w*(*n*) is a window function with the window size *L*, L∈{64, 128, 256, 512}  and R is the hop length R∈{20, 30, 40, 50}. The window size, *L*, represents the number of samples included in each window when computing the fast Fourier transform [32]. Both the window size and the hop length determine how the spectrogram represents the sound data. Generally, the window size is relevant to the frequency resolution and the time resolution of the spectrogram. These two parameters were selected to extract the best features for DS-CNN. Figure 2 demonstrates the continuous-sound, discontinuous-sound, and normal-sound spectrograms.

#### 2.2.2. MFCC Feature

On the basis of cepstrum analysis, the Mel-frequency cepstrum analysis was developed, where the human auditory system’s response to sounds was considered. The relation between the Mel-frequency, m, and the frequency, f, is defined as

(2) m=2595 log(1+f/700).

The spectrums windowed by equally spaced Mel-frequency seem to cause comparable sensitivities for human auditory perception, and this motivates the usage of MFCC, which is derived through the following steps [33]:

(1) Calculate the power spectrum, ${\left|\underline{X}\left(f\right)\right|}^{2},$ of the sound signal, x(t), through Fourier transform.
(2) Map a set of equally spaced Mel-frequencies, $\left\{m_{k},\;k=1,2,3\ldots \right\}$, to the frequency domain to obtain $\left\{f_{k},\;k=1,2,3\ldots \right\}.$
(3) Use the triangular windows centered at $\left\{f_{k}\right\}$ to get the weighted sum of the power spectrum and then take the logarithm of the power integral for each Mel-frequency
(4) Use discrete cosine-transform to transform the logarithmic power to get MFCCs.

**Figure 3:**
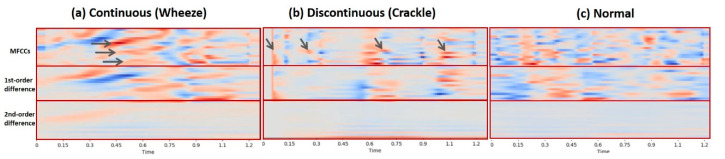
The MFCC features of the continuous sound, discontinuous sound, and normal sound are visualized in (**a**–**c**), respectively. The arrows in (**a**) point at the dark red areas where the coefficients are positive. Those dark red areas tend to form irregular texture patterns. The arrows in (**b**) indicate that the dark red areas alternate with the blue areas where the coefficients are negative, which tends to form vertical-stripe-like patterns.

This paper adopted a short-time version of MFCC, where a period of time signal was taken to extract the MFCC feature. The first-order and second-order differences of MFCCs were also extracted and appended to MFCCs as one MFCC feature. The number of MFCC coefficients, N_mfcc, $N_{\mathrm{mfcc}}\in \left\{10,\;13,\;20\right\},\;$ was selected as a parameter for tuning the appropriate feature. Figure 3 shows the MFCC features of continuous, discontinuous, and normal lung sounds.

### 2.3. DS-CNN

**Figure 4:**
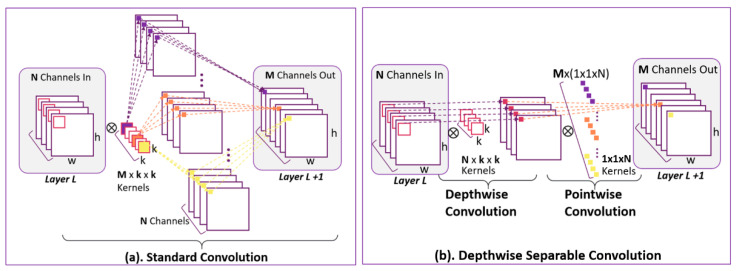
(**a**) Standard convolution (**b**) Factorizing standard convolution into depthwise convolution and pointwise convolution.

Factorizing standard convolution into depthwise convolution and pointwise convolution is the key to accelerating convolution operations for DS-CNN. Figure 4 describes how the standard and depthwise separable convolution work.

In what follows, we explicitly compare the computation costs between DS-CNN and standard CNN layers. Considering the convolutional operation, which is assumed stride one, padding same, and applied on layer *L* in a neural network, the computational cost of standard convolution in Figure 4a is

(3) w · h · N · k · k · M,

where *w*, *h*, and *N* are the width, height, and channel number of the input feature map at layer *L*, respectively. *M* is the number of square convolution kernels with *k* spatial dimensions. For DS CNN in Figure 4b, the computational cost of depthwise convolution is

(4) w · h · N · k · k .

The computational cost of pointwise convolution is

(5) w · h · 1 · 1 · N · M ,

where *N* is the depth of the 1 × 1 convolution kernel, which combines *N* channels’ features produced by depthwise convolution. *M* is the number of 1 × 1 × *N* convolution kernels to produce *M* output feature maps at layer *L* + 1 with width, *w*, and height, *h*. The reduction in computation by factorizing standard convolution into depthwise convolution and pointwise convolution is

(6) $$\frac{computational\;cost\;of\;DS-CNN}{computational\;cost\;of\;stand\;CNN}\;=\frac{w\;\cdot \;h\;\cdot \;N\;\cdot \;k\;\cdot \;k+w\;\cdot \;h\;\cdot \;1\;\cdot \;1\;\cdot \;N\;\cdot \;M}{w\;\cdot \;h\;\cdot \;N\;\cdot \;k\;\cdot \;k\;\cdot \;M}\;=\frac{1}{M}+\frac{1}{k^{2}\;.}\;$$

#### Shrinking DS-CNN Model

To shrink the model and retain the model performance, a model selection procedure was derived from reference [29]. The width multiplier, α, α ∈ {0.75, 0.5}, and the number of DS blocks, β, β ∈ {12, 10, 8}, were adopted to form a simple 2 × 3 grid for model selection. The architecture of the original MobilNet, including 13 DS-blocks and approximately 2 million parameters, was taken as the reference model. The width multiplier, α, was used to determine the width of DS-CNN by evenly reducing the number of convolution kernels or fully connected nodes for each layer. The reduced numbers of convolution kernels were calculated by multiplying α with the original number of convolution kernels. The number of DS blocks, β, was used to determine the depth of DS-CNN. The numbers of parameters of different shrunk models produced by the combinations of α and β are listed in Table 1.

Eventually, the model with α = 0.75 and β = 10 was selected to strike a balance between model performance and model complexity. The DS-CNN model was trained from scratch without pre-trained weight. No data augmentation techniques were applied to model training.

### 2.4. Model Evaluation

The models were evaluated and compared in a hierarchical way as follows:

Level 1: comparison among features
Level 2: comparison among deep learning model architectures
Level 3: comparison between our method and the other method

In level 1 comparison, the best features were selected through the feature engineering process. The performances of a total of three features, the STFT feature, the MFCC feature, and the fused features of these two, were compared.

In level 2 comparison, the performances of DS-CNN, standard-CNN, and RNN were compared. Vgg16 [34], AlexNet [35], DS-AlexNet, Long Short-Term Memory (LSTM) [36], Gated Recurrent Unit (GRU) [37], and Temporal Convolutional Network (TCM) [38] were selected for comparison with DS-CNN. The selected models were trained using the fused features of STFT and MFCC.

In level 3 comparison, RespireNet [22] was selected as the baseline to evaluate our method because RespireNet is open source, which can be reproduced exactly like the original way of implementation. On the contrary, the other methods [19,21,25] without the publicly released codes were not selected for comparison. The best model of our method and RespireNet were converted to TensorFlow Lite (TF Lite) models to accelerate model inferencing. Eighty respiratory cycles, which contained 20 cycles of each lung sound type, were selected for measuring the inference time. The inference time included the time of feature extracting and model inferencing. The inference times of our method and RespireNet were compared on both the edge device, Raspberry Pi 3 B+, and the cloud server, Google Colab (CPU runtime), with TF Lite models.

## 3. Results

The models’ performances were evaluated by the index of F1 score, recall, precision, and accuracy. For each sound type

i∈{Continuous, Discontinuous, Normal, Unknown}

(7) $$Recall_{}\;REC=\frac{M\left[i,\;i\right]}{\sum_{j}^{}M\left[i,j\right]}$$

(8) $$Precision_{}\;PRC=\frac{M\left[i,\;i\right]}{\sum_{j}^{}M\left[j,i\right]}$$

(9) $$F1\;score_{}\;F1=\frac{2*PRC\;*\;REC}{PRC+REC}.\;$$

Here an element, *M*[*i*,*j*], of the 4 × 4 confusion matrix, *M*, indicates that *M*[*i*,*j*] samples are predicted to be label *j* but are indeed label *i*. The overall accuracy is defined as

(10) $$\mathrm{Accuracy}=\frac{\sum_{i}^{}M\left[i,i\right]}{\sum_{i,j}^{}M\left[i,j\right]}.$$

The results of level 1 to level 3 comparison examined our method’s performance across features, model architecture, and levels of inference efficiency on edge devices. Table 2 shows the results of level 1 comparison. In level 1 comparison, the best STFT feature was extracted when the window size and the hop length were 512 and 40, respectively. The best MFCC feature was extracted when the number of MFCCs was 20. The fused features of STFT and MFCCs, which performed the best, were extracted when the windows size and the hop length were 256 and 40, respectively, after fine-tuning. According to Table 2, all the indexes, including precision, recall, F1 score, and accuracy, were substantially increased when STFT and MFCCs were fused as one feature.

Table 3 summarizes the results of level 2 comparison. In level 2 comparison, CNN-based models outperformed RNN-based models. Also, the shrunk DS-CNN model achieved higher accuracy than standard CNN models. The shrunk DS-CNN model with only 1.36 million parameters achieved the best accuracy, 85.74%. The second-best accuracy, 85.66%, was yielded by VGG-16 with 67.03 million parameters.

The results of level 3 comparison are shown in Table 4 and Table 5. According to Table 4, our method performed nearly as accurately as RespireNet did. Our F1 scores of continuous and discontinuous are equal to RespireNet’s, which are 0.89 and 0.82, respectively. Our method achieved 85.74% accuracy, only 0.43% less than RespireNet, which achieved 86.17%. On the contrary, our method had 16 times higher inference speed and 16 times smaller model size than RespireNet on the edge device, according to Table 5.

**Figure 5:**
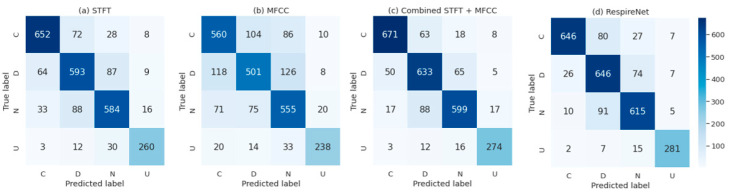
Confusion matrices of (**a**) DS-CNN trained with STFT feature, (**b**) DS-CNN trained with MFCC feature, (**c**) DS-CNN trained with fused STFT and MFCC features, and (**d**) RespireNet. Continuous, discontinuous, normal, and unknown are abbreviated as C, D, N, and U, respectively.

The confusion matrices of level 1 and level 3 comparisons are shown in Figure 5. For level 1 comparison in Figure 5a–c, the DS-CNN trained with fused STFT and MFCC features had higher correct predictions for each lung sound type than the other two models. For level 2 comparison in Figure 5b,c, our method’s confusion matrix presented a trend similar to that of RespireNet.

## 4. Discussion

**Figure 6:**
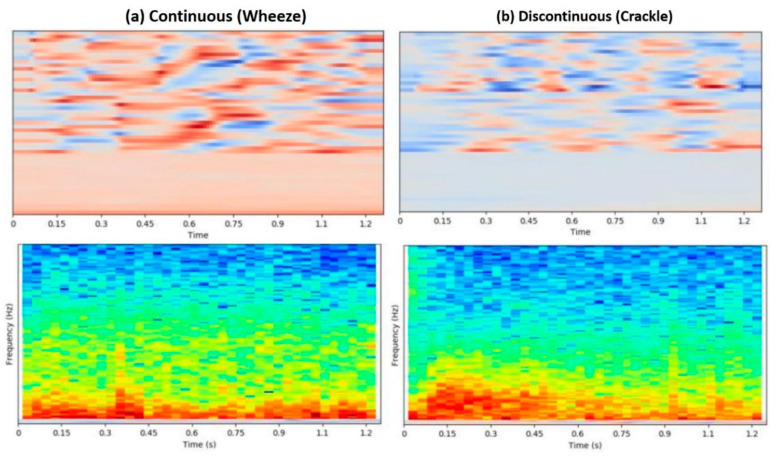
The upper part and the lower part show the MFCC feature and the STFT feature, respectively. The STFT feature of (**a**) continuous and (**b**) discontinuous sounds shows few distinctions between the two lung sound types. On the contrary, the MFCC feature appears to be distinguishable between the two. The STFT feature and the MFCC feature tend to be complementary to each other.

The shrunk DS-CNN model performance substantially increased when the model was trained with the fused features of STFT and MFCC. The STFT and MFCC features may complement each other because the MFCC feature represents human auditory perception more closely. Therefore, some acoustic distinctions between different types of lung sounds may be enhanced by the MFCC feature. Figure 6 shows an example of the situation mentioned earlier. Besides, the feature should also be extracted with only a few computational costs to take advantage of DS-CNN, which accelerates convolution operations to a great extent on edge devices. Both STFT and MFCC can be calculated efficiently by the fast Fourier transform algorithm [32] to avoid the bottleneck in the feature extraction step.

The fused features of STFT and MFCC extracted from the proposed feature engineering process contributed to the shrunk DS-CNN model’s high accuracy compared with model architectures. Moreover, all CNN-based models outperformed RNN-based models in terms of accuracy. The results of level 2 comparison indicate that the fused features that we extracted are appropriate for DS-CNN-based models. CNN-based models were originally designed for image recognition tasks, whereas RNN-based models were designed for learning the features of sequences. The STFT and MFCC features can resemble either images or multi-dimensional time-series data. However, we fine-tuned the fused features based on DS-CNN-based models rather than RNN-based models. There is inevitably a trade-off between frequency resolution and time resolution when extracting STFT and MFCC features. The demand for frequency or time domain resolution may depend on the model architectures. Hence, the appropriate features for DS-CNN-based models may not have enough time domain resolution for the RNN-based models. Additionally, the proposed feature engineering process can be employed to extract the appropriate features for any other model architectures. Likewise, the lung sound can be replaced by other sound types, such as heart sounds.

Compared with RespireNet, our method provided a smaller-sized model, higher inference speed, and comparable model performance. This result presents a trend similar to the study of respiratory sound classification in wearable devices [19]. As observed in reference [19], the DS-CNN-based model (MobileNet) required the least computational complexity and had only 4.78% less F1 score than the best model they proposed on the ICBHI dataset. When it comes to developing the automatic lung sound recognition system on edge devices, the models should not consume too much computational power and memory space. There should be enough hardware resources to maintain the operations of the whole system. Through the proposed feature engineering and model-shrinking process, a shrunk DS-CNN model may be trained to recognize lung sounds on edge devices accurately and efficiently.

The model training process adopted by the original RespireNet is consistent with many previous studies [19,21,25]. They used the ICBHI dataset, pre-trained weights, and used augmented data to train their CNN-based models. The sound signals were transformed into 3-channel color images. Those color images were preprocessed by cropping or resizing to enhance visual patterns for the model to learn features. However, our method used original values of STFT spectrograms and MFCCs with only one channel rather than three channels to train all CNN-based models. We expected the model to learn the features that reveal the direct and intuitive information of the spectrograms. The CNN-based models were trained from scratch without pre-trained weights and data augmentation because the dataset used in this research is different and larger than the ICBHI dataset. The results shown in Table 4 imply that the DS-CNN model may learn the features from original spectrograms without pre-trained weights if the dataset is large enough.

Furthermore, a possible explanation for our method achieving lower evaluation indexes of the unknown lung sound might be that there is no data augmentation adopted through model training. The unknown lung sound dataset is not as large as any other three types of lung sound dataset. The data augmentation technique originally proposed by RespireNet to handle the data imbalance issue of the ICBHI dataset may lead to better performance for recognizing the unknown lung sounds.

Autonomous stethoscopes developed by integrating AI-algorithm into portable digital stethoscopes have been proposed by Glangetas et al. [39]. Portable digital stethoscopes can be various forms of smartphone accessories for easy mobility [40]. The fused STFT, MFCC features, and DS-CNN model may be one appropriate AI algorithm for autonomous stethoscopes. The autonomous stethoscopes appear to increase the accessibilities of high-quality lung auscultation to medical workers or patients for self-monitoring. With the help of this device, clinicians and caregivers could interpret pathological and physiological information in the lung sounds at the first sign of a patient’s abnormal conditions. This information tends to be practical to identify the need for timely treatment or early hospitalization.

## 5. Conclusions

We have proposed a feature engineering process to extract dedicated features for the shrunk DS-CNN to classify four types of lung sounds. We observed that fusing the STFT and MFCC features led to a higher accuracy of 85.74%. In contrast, the model trained on only one STFT or MFCC feature achieved the accuracies of 82.27% and 73.02%, respectively. We then evaluated our method by comparing it with RespireNet. While RespireNet was 0.43% better than our method in terms of accuracy, our method achieved 16 times higher inference speed on the edge device.

To summarize, these results support the idea that DS-CNN may perform nearly as accurately as standard CNN by training with appropriate features. The feature engineering process that we have proposed can be applied to the extraction of dedicated features for other types of sound signals or for other architectures of deep learning models. However, we did not use any data augmentation techniques in this study. Further research might explore how data augmentation techniques affect the performance of sound recognition models.

## Figures and Tables

**Table 1:** The numbers of million parameters of different shrunk models produced by combinations of α and β.

	Depth	12-DS Blocks β = 12	10-DS Blocks β = 10	8-DS Blocks β = 8
Width				
α = 0.75		1.67	**1.36**	1.05
α = 0.50		0.76	0.61	0.47

The number of million parameters in bold indicates that the shrunk model was finally selected.

**Table 2:** Results of level 1 comparison.

	STFT			MFCC			Fused Features		
	F1	REC	PRC	F1	REC	PRC	F1	REC	PRC
Continuous	0.86	0.86	0.87	0.73	0.74	0.73	0.89	0.88	0.91
Discontinuous	0.78	0.79	0.78	0.69	0.67	0.72	0.82	0.84	0.80
Normal	0.81	0.81	0.80	0.73	0.77	0.69	0.84	0.83	0.86
Unknown	0.87	0.85	0.89	0.82	0.78	0.86	0.90	0.90	0.90
Accuracy	82.27%			73.02%			85.74%		

**Table 3:** Results of level 2 comparison.

	Model.	DS-CNN *	VGG-16	AlexNet	DS-AlexNet	LSTM	GRU	TCN
Lung Sound Types								
		**F1 Score**						
Continuous		0.89	0.89	0.85	0.85	0.81	0.80	0.78
Discontinuous		0.82	0.81	0.77	0.75	0.69	0.73	0.70
Normal		0.84	0.84	0.77	0.78	0.75	0.78	0.74
Unknown		0.90	0.91	0.79	0.89	0.88	0.88	0.86
Accuracy		85.74%	85.66%	79.92%	80.86%	76.92%	78.50%	75.51%
Million Parameters		1.36	67.03	32.99	1.71	0.29	0.23	0.02

* DS-CNN means the shrunk DS-CNN model.

**Table 4:** Results of level 3 comparison-1: Model performance.

	Our Method			RespireNet		
	F1	REC	PRC	F1	REC	PRC
Continuous	0.89	0.88	0.91	0.89	0.85	0.94
Discontinuous	0.82	0.84	0.80	0.82	0.86	0.78
Normal	0.84	0.83	0.86	0.85	0.85	0.84
Unknown	0.90	0.90	0.90	0.93	0.92	0.94
Accuracy	85.74%			86.17%		

**Table 5:** Results of level 3 comparison-2: Model Inference.

	Comparison	Inference Time per Cycle on Edge	Inference Time per Cycle on Cloud	Model Architecture	Million Parameters	TF Lite Model Size
Method						
Our Method		0.22 s	0.038 s	DS-CNN	1.36	5 MB
RespireNet		3.54 s	0.45 s	Resnet 34	21.36	81 MB
